# The Cellular and Developmental Roles of Cullins, Neddylation, and the COP9 Signalosome in *Dictyostelium discoideum*

**DOI:** 10.3389/fphys.2022.827435

**Published:** 2022-03-01

**Authors:** William D. Kim, Sabateeshan Mathavarajah, Robert J. Huber

**Affiliations:** ^1^Environmental and Life Sciences Graduate Program, Trent University, Peterborough, ON, Canada; ^2^Department of Pathology, Dalhousie University, Halifax, NS, Canada; ^3^Department of Biology, Trent University, Peterborough, ON, Canada

**Keywords:** COP9 signalosome, cullins, *Dictyostelium discoideum*, neddylation, SCF complex

## Abstract

Cullins (CULs) are a core component of cullin-RING E3 ubiquitin ligases (CRLs), which regulate the degradation, function, and subcellular trafficking of proteins. CULs are post-translationally regulated through neddylation, a process that conjugates the ubiquitin-like modifier protein neural precursor cell expressed developmentally downregulated protein 8 (NEDD8) to target cullins, as well as non-cullin proteins. Counteracting neddylation is the deneddylase, COP9 signalosome (CSN), which removes NEDD8 from target proteins. Recent comparative genomics studies revealed that CRLs and the CSN are highly conserved in Amoebozoa. A well-studied representative of Amoebozoa, the social amoeba *Dictyostelium discoideum*, has been used for close to 100 years as a model organism for studying conserved cellular and developmental processes owing to its unique life cycle comprised of unicellular and multicellular phases. The organism is also recognized as an exceptional model system for studying cellular processes impacted by human diseases, including but not limited to, cancer and neurodegeneration. Recent work shows that the neddylation inhibitor, MLN4924 (Pevonedistat), inhibits growth and multicellular development in *D. discoideum*, which supports previous work that revealed the cullin interactome in *D. discoideum* and the roles of cullins and the CSN in regulating cellular and developmental processes during the *D. discoideum* life cycle. Here, we review the roles of cullins, neddylation, and the CSN in *D. discoideum* to guide future work on using this biomedical model system to further explore the evolutionarily conserved functions of cullins and neddylation.

## Neddylation, Cullins, and the COP9 Signalosome

**Figure 1:**
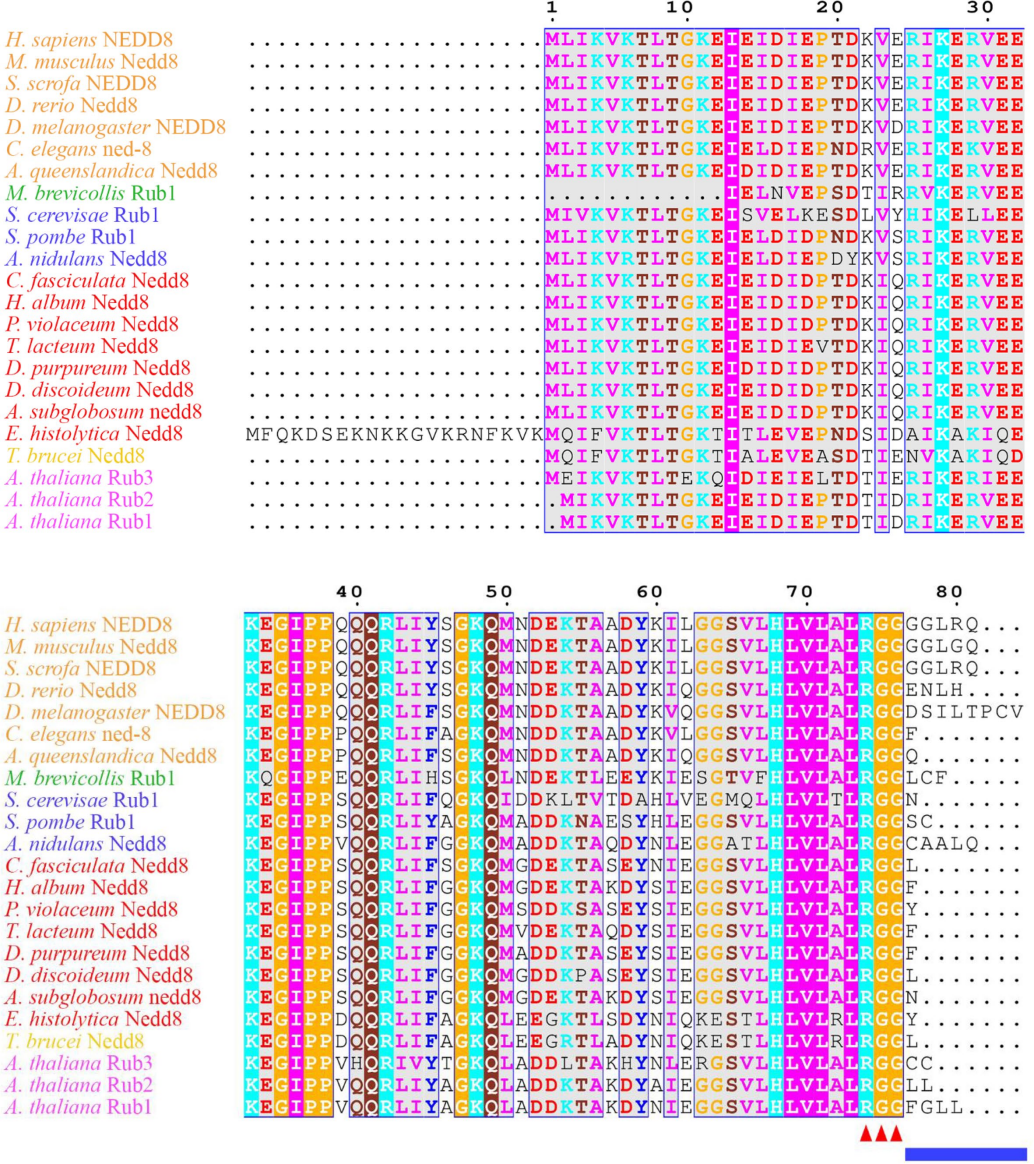
Neural precursor cell expressed developmentally downregulated protein 8 (NEDD8) is conserved across eukaryotes. Alignment of NEDD8 ortholog protein sequences in different eukaryote species, including those in plants (pink), amoebozoa (red), fungi (blue), and animals (orange). For plants, the upstream ubiquitin sequence was trimmed from the NEDD8 orthologs. Sequences were aligned on MEGA7 using the MUSCLE alignment and displayed using ESPript 3.0. Colored residues indicate physiochemical properties and conservation at a position. The C-terminal “RGG” proteolytic processing motif is marked by red arrowheads and the NEDD8 overhang sequence that is cleaved is indicated by the blue line.

The continual turnover of proteins through degradation maintains cell homeostasis, facilitates signal transduction, and allows for progression through the cell cycle. One of the pathways cells use to degrade proteins involves the proteasome, where ubiquitin is the tag that marks proteins for degradation. In addition to ubiquitin, there are also ubiquitin-like modifiers that target both proteins and lipids to control their subcellular localization, macromolecular interactions, and enzymatic activity (Cappadocia and Lima, 2018). Known ubiquitin-like modifiers include small ubiquitin-like modifier (SUMO), ubiquitin fold modifier 1 (UFM1), ubiquitin-related modifier 1 (URM1), ubiquitin-like modifier HUB1, and neural precursor cell expressed developmentally downregulated protein 8 (NEDD8; Vierstra, 2012). NEDD8 is highly conserved across eukaryotes (Figure 1) and is conjugated to target proteins at a near-terminal lysine residue (N-term or C-term) through a process known as neddylation. The modification was first observed in the *Saccharomyces cerevisiae* S phase kinase-associated protein 1 (Skp1)-Cullin-F-box (SCF) complex, where Rub1, the *S. cerevisiae* ortholog of human NEDD8, was found conjugated to Cdc53p, the *S. cerevisiae* ortholog of cullin 1 (CUL1; Lammer et al., 1998; Liakopoulos et al., 1998). In the SCF complex, the linker protein SKP1 bridges the interaction between the cullin and F-box protein, which targets specific substrates for ubiquitination (Zimmerman et al., 2010). Cullins then serve as the scaffold for multi-subunit ubiquitin ligases. SCF is a member of the cullin–RING E3 ubiquitin ligase (CRL) superfamily, which plays important roles in regulating a variety of proteins including transcription factors, cell cycle regulators, DNA damage response/repair proteins, and growth factor receptors (Schwechheimer and Villalobos, 2004; Vodermaier, 2004; Willems et al., 2004; Ardley and Robinson, 2005; Bosu and Kipreos, 2008; de Bie and Ciechanover, 2011; Li and Jin, 2012; Chung and Dellaire, 2015; Qi and Ronai, 2015; Gilberto and Peter, 2017; Serrano et al., 2018). In addition, neddylation plays an important role in regulating the activity of subunits that form the proteasome and ribosomes (Xirodimas et al., 2008). During proteotoxic stress, neddylation promotes ribosomal protein accumulation in the nucleus to protect the proteasome system and prevent dysfunction (Maghames et al., 2018). During oxidative stress, neddylation regulates poly(ADP-ribose) polymerase 1 (PARP1) activity to delay the initiation of PARP1-dependent cell death (Keuss et al., 2019). Thus, neddylation has multiple essential functions in the cell.

**Figure 2:**
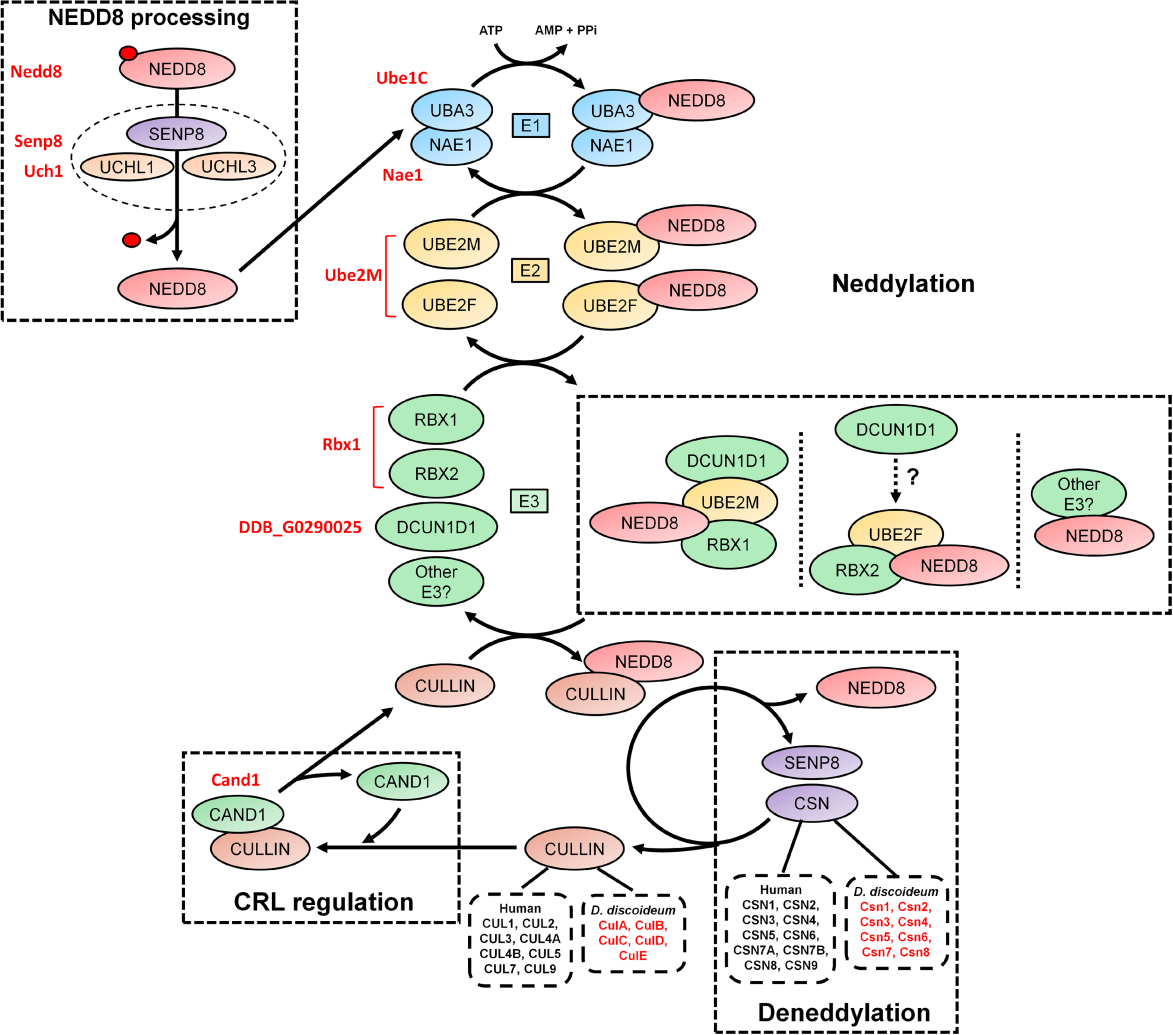
An overview of the neddylation and deneddylation pathway. NEDD8 is processed into its matured form through both ubiquitin C-terminal hydrolase isozyme (UCH) and Sentrin-specific protease 8 (SENP8) proteolytic cleavage. NEDD8 is passed through a “baton” mechanism, where NEDD8 is adenylated and activated in an adenosine triphosphate (ATP)-dependent mechanism by E1, which is a heterodimer of NEDD8-activating enzyme E1 regulatory subunit (NAE1) and ubiquitin-activating enzyme 3 (UBA3). NEDD8 binds specifically to UBA3 within E1. UBA3 binds both ubiquitin-conjugating enzyme (UBE) 2F (UBE2F) and ubiquitin-conjugating enzyme E2M (UBE2M) in E2 and transfers NEDD8 to both proteins. NEDD8 is finally attached to a substrate (i.e., cullins and non-neddylated proteins) by E3, which includes ring box 1 (RBX1), ring box 2 (RBX2), and other potential E3 ligases. In E3, NEDD8 loaded into UBE2M associates with RBX1 and defective in cullin neddylation 1 (DCUN1D1). NEDD8 loaded into UBE2F associates with RBX2, but the involvement of DCUN1D1 is unknown. Substrate neddylation displaces cullin-associated NEDD8-dissociated protein 1 (CAND1), which serves to regulate cullin-RING E3 ubiquitin ligases (CRL) assembly. NEDD8 is removed from the substrate via deneddylation, which involves the COP9 signalosome (CSN) complex and SENP8/DEN1. The *Dictyostelium discoideum* orthologs are displayed as red text beside the respective human protein. AMP, adenosine monophosphate; ATP, adenosine triphosphate; CAND1, cullin-associated NEDD8-dissociated protein 1; CSN, COP9 signalosome; CUL, cullin; E1, E1-NAE1 complex; E2, E2-conjugation complex; E3/CRL, E3-cullin-RING complex; NAE1, NEDD8 activating enzyme E1 subunit 1; NEDD8, neural precursor cell expressed developmentally downregulated protein 8; PPi, pyrophosphate; RBX, ring box; SENP8, sentrin-specific protease 8; UBA3, ubiquitin-activating enzyme 3; UBE, ubiquitin-conjugating enzyme; UCH, ubiquitin C-terminal hydrolase; and UCHL, ubiquitin C-terminal hydrolase isozyme.

Many aspects of neddylation mirror and incorporate pathways that are associated with ubiquitination. Akin to ubiquitin, once NEDD8 is translated into an inactive precursor form, it requires cleavage of its short C-terminal amino acid extension (five amino acids in humans) to generate the mature form of the protein (Figures 1, 2; Kamitani et al., 1997). Mature NEDD8 has an accessible C-terminal glycine residue positioned at amino acid 76 that is used to conjugate NEDD8 to the lysine of a target protein (Figure 1; Kamitani et al., 1997). NEDD8 C-terminal cleavage occurs through the actions of ubiquitin C-terminal hydrolase isozyme (UCH) L1 (UCHL1) and UCHL3 (both belonging to the C12 family of peptidases; Figure 2). UCHs also cleave the C-terminal extensions of ubiquitin (Wada et al., 1998; Johnston et al., 1999; Linghu et al., 2002; Hemelaar et al., 2004; Frickel et al., 2007; Yu et al., 2011). While UCHL1 and UCHL3 both bind to NEDD8, the hydrolytic processing of NEDD8 is carried out by UCHL3 (Wada et al., 1998). Sentrin-specific protease 8 (SENP8/DEN1; belonging to the C48 family of peptidases) is another NEDD8 processing enzyme that exclusively targets NEDD8 and not ubiquitin (Figure 2; Gan-Erdene et al., 2003; Mendoza et al., 2003; Wu et al., 2003; Shen et al., 2005; Chan et al., 2008; Shin et al., 2011). Like UCHL3, SENP8 also cleaves NEDD8 at the 5-amino acid C-terminal extension suggesting the functions of the two enzymes are redundant (Mendoza et al., 2003).

Like ubiquitination, the conjugation of mature NEDD8 to target proteins follows an E1-E2-E3 cascade (Figure 2; Kawakami et al., 2001; Pan et al., 2004; Huang et al., 2005; Saha and Deshaies, 2008). After NEDD8 is cleaved, it is adenylated and activated by E1 in an adenosine triphosphate (ATP)-dependent mechanism (Bohnsack and Haas, 2003; Walden et al., 2003). E1 is a heterodimer of NEDD8-activating enzyme E1 regulatory subunit (NAE1) and ubiquitin-activating enzyme 3 (UBA3; Gong and Yeh, 1999; Bohnsack and Haas, 2003; Walden et al., 2003). Within E1, NEDD8 resides between NAE1 and UBA3 but binds directly with the latter. E1 then transfers NEDD8 to E2, which is comprised of ubiquitin-conjugating enzyme (UBE) 2F (UBE2F) and UBE2M (Osaka et al., 1998; Gong and Yeh, 1999; Huang et al., 2009). Both UBE2F and UBE2M can bind NEDD8 (Gong and Yeh, 1999; Huang et al., 2005, 2009). E2 receives assistance in the final step of neddylation from a few E3 ligases such as ring box 1/regulator of cullins 1 (RBX1/ROC1), ring box 2/regulator of cullins 2 (RBX2/ROC2), and defective in cullin neddylation 1 (DCUN1D1), to transfer NEDD8 to the lysine of target proteins (e.g., cullins) and initiate CRL assembly (Kamura et al., 1999; Petroski and Deshaies, 2005; Duda et al., 2008; Saha and Deshaies, 2008; Huang et al., 2009; Scott et al., 2010). Previous work showed that RBX1 and DCUN1D1 interact with the NEDD8-UBE2M intermediate to neddylate CUL1, CUL3, CUL3, and CUL4 (Kim et al., 2008; Huang et al., 2009). CUL5 neddylation is carried out by RBX2 in association with UBE2F, but unlike RBX1, it is not known if DCUN1D1 also participates in the conjugation (Huang et al., 2009). Finally, CUL7 and CUL9 have been shown to bind to RBX1 (Andrews et al., 2006). Like ubiquitination, proteins can be polyneddylated (Jones et al., 2008). However, unlike ubiquitin, which has over 10,000 targets in humans, NEDD8 appears to be conjugated to a shorter list of proteins. For example, Jones et al. (2008) performed a targeted proteomics analysis to identify 496 NEDD8-modified and associated proteins in HEK293 cells, which included all human cullins. In addition, a recent study identified 1,101 unique neddylation sites on 620 human proteins in HEK293 cells (Lobato-Gil et al., 2021). Cullins undergo neddylation at a consensus C-terminal neddylation motif [(IL)(VIT)(RQ)(IS)(MLV)K(MAS)(RHE)] and are conjugated specifically to a lysine residue found within the motif (Mikus and Zundel, 2005). Therefore, neddylation represents a unique but smaller pool of ubiquitin-like modification within the eukaryotic cell, where cullins are the major targets.

The dynamic assembly of CRL complexes is modulated by cullin-associated NEDD8-dissociated protein 1 (CAND1), which functions as a SKP1/F-box protein exchange factor for CUL1 (as well as other cullins; Figure 2; Zheng et al., 2002; Dubiel et al., 2013; Pierce et al., 2013; Liu et al., 2018). Neddylation of CUL1 and subsequent binding of SKP1 and a F-box protein causes the displacement of CAND1, thus allowing for CRL assembly (Liu et al., 2002; Zheng et al., 2002). Without the incorporation of CAND1, there is inefficient degradation of target proteins. Neddylation also contributes to the enzymatic activity of the SCF complex by causing a conformational shift to improve ubiquitin transfer activity and E2 recruitment (Saha and Deshaies, 2008). The important role of this process is highlighted in studies that showed that the complete loss of neddylation is lethal (with *S. cerevisiae* being the exception; Lammer et al., 1998; Osaka et al., 2000; Tateishi et al., 2001). In humans, abnormalities in neddylation are linked to a variety of pathological conditions including cancer, neurodegeneration, autoimmune diseases, and other inflammatory diseases (Chen et al., 2012; Enchev et al., 2015; Ehrentraut et al., 2016; Ying et al., 2018). Together, these findings suggest that neddylation is a key component of CRL regulation, and when aberrant, contributes to the pathogenesis associated with many human diseases.

Deneddylation (removal of NEDD8 from proteins) occurs through the actions of the COP9 signalosome (CSN), which is composed of nine subunits in humans (CSN1-6, CSN7A/7B, and CSN8-9; Figure 2; Rao et al., 2020). SENP8/DEN1, which participates in NEDD8 processing (discussed above), also plays a role in disassembling CRLs (Wu et al., 2003; Shin et al., 2011). The CSN exists as two variant complexes containing CSN1-6, CSN8-9, and one of CSN7A or CSN7B, which have overlapping functions in the deneddylation of CRLs (Wang et al., 2021). However, CSN7B has been reported to have a unique function in adipogenesis and the DNA damage response (Huang et al., 2016; Wang et al., 2021). The CSN is conserved across eukaryotes including plants (e.g., *Arabidopsis thaliana*), invertebrates (e.g., *Drosophila melanogaster* and *Caenorhabditis elegans*), yeast (e.g., *S. cerevisiae* and *Schizosaccharomyces pombe*), fungi (e.g., *Neurospora crassa* and *Aspergillus nidulans*), and humans (Schwechheimer et al., 2001; Busch et al., 2003; Doronkin et al., 2003; Pintard et al., 2003; He et al., 2005; Wee et al., 2005; Wu et al., 2005; Cope and Deshaies, 2006; Schmidt et al., 2010). CSN complexes that contain fewer than nine subunits have been observed in different eukaryotic clades, suggesting that the protein architecture of the complex has been prone to changes over time (Braus et al., 2010). However, a consistent hallmark is the conservation of the CSN5 subunit among different eukaryotes.

As discussed above, the CSN subunits exhibit widespread abundance throughout both unicellular and multicellular eukaryotes (Barth et al., 2016). Amoebozoan genomes encode either a CSN with all the known subunits or all but CSN8 and CSN9 subunits (Barth et al., 2016). A representative model organism from Amoebozoa is the social amoeba, *Dictyostelium discoideum*, which was identified as a species with a genome encoding an intact eight subunit CSN (Rosel and Kimmel, 2006; Heidel et al., 2011). *D. discoideum* emerged at least 600 million years ago (an amorphea that diverged prior to the fungi-animal split) and has been studied for close to a century (Mathavarajah et al., 2017). Various cullins have been identified as key regulators of multicellular development in *D. discoideum* (Mohanty et al., 2001; Wang and Kuspa, 2002; Sheikh et al., 2015). Furthermore, components of the neddylation pathway in metazoans are conserved in *D. discoideum* (Figure 2; Table 1), and there is evidence supporting cullin neddylation during the life cycle (Sheikh et al., 2015). Here, we review the known and predicted roles of cullins and neddylation in *D. discoideum* to set the stage for future work that further examines how cullins and neddylation regulate conserved cellular and developmental processes.

**Table 1:** Sequence similarity between neddylation pathway proteins and CSN subunits in humans and *Dictyostelium discoideum*.

Human protein (Uniprot ID)	Size (aa)	*Dictyostelium discoideum* ortholog (dictyBase gene ID)	Size (aa)	Region of similarity (aa)	Identities (%)^*^	Positives (%)^**^	E-value
**NEDD8 processing**							
NEDD8 (Q15843)	81	Nedd8 (DDB_G0278711)	77	76	82	92	9E-26
SENP8/DEN1 (Q96LD8)	212	Senp8 (DDB_G0278795)	243	241	32	47	4E-26
UCHL1 (P09936)	223	Uch1 (DDB_G0282007)	255	221	47	66	3E-54
UCHL3 (P15374)	230			224	52	70	1E-65
**NAE1 heterodimer**							
NAE1/APPBP1 (Q13564)	534	Nae1 (DDB_G0287965)	520	524	41	64	1E-123
UBA3 (Q8TBC4)	463	Ube1c (DDB_G0283891)	442	441	49	68	1E-128
**E2**							
UBE2M/UBC12 (P61081)	183	Ube2M (DDB_G0281725)	230	178	53	73	1E-54
UBE2F (Q969M7)	185			175	33	53	6E-24
**E3**							
RBX1/ROC1 (P62877)	108	Rbx1 (DDB_G0287629)	104	97	86	92	2E-42
RBX2/ROC2 (Q9UBF6)	113			104	46	60	4E-20
DCUN1D1 (Q96GG9)	259	Unnamed (DDB_G0290025)	249	253	37	56	6E-43
**Regulator of CRL assembly**							
CAND1 (Q86VP6)	1,230	Cand1 (DDB_G0274167)	1,238	1,256	40	61	0
**CSN subunits**							
CSN1/COPS1/GPS1 (Q13098)	491	Csn1 (DDB_G0283587)	458	436	45	63	1E-102
CSN2/COPS2/TRIP15 (P61201)	443	Csn2 (DDB_G0289361)	449	417	63	79	1E-143
CSN3/COPS3 (Q9UNS2)	423	Csn3 (DDB_G0291848)	418	379	40	61	2E-76
CSN4/COPS4 (Q9BT78)	406	Csn4 (DDB_G0293844)	393	387	47	71	9E-95
CSN5/COPS5/JAB1 (Q92905)	334	Csn5 (DDB_G0284597)	332	332	66	81	1E-130
CSN6/COPS6/HVIP (Q7L5N1)	327	Csn6 (DDB_G0293180)	309	288	41	64	2E-61
CSN7A/COPS7A/DERP10 (Q9UBW8)	275	Csn7 (DDB_G0271282)	259	194	41	68	1E-43
CSN7B/COPS7B (Q9H9Q2)	264			182	46	69	1E-41
CSN8/COPS8 (Q99627)	209	Csn8 (DDB_G0275471)	196	200	30	54	1E-24
CSN9/COPS9 (Q8WXC6)	57	Could not be identified	-	-	-	-	-

BLASTp searches were performed using dictyBase. Human proteins were used as query sequences (E-value, 0.1; Matrix, BLOSUM62; Filter, no).

* Exact amino acid match.

** Similar amino acid match (e.g., both polar).

## The Life Cycle of *Dictyostelium discoideum*

**Figure 3:**
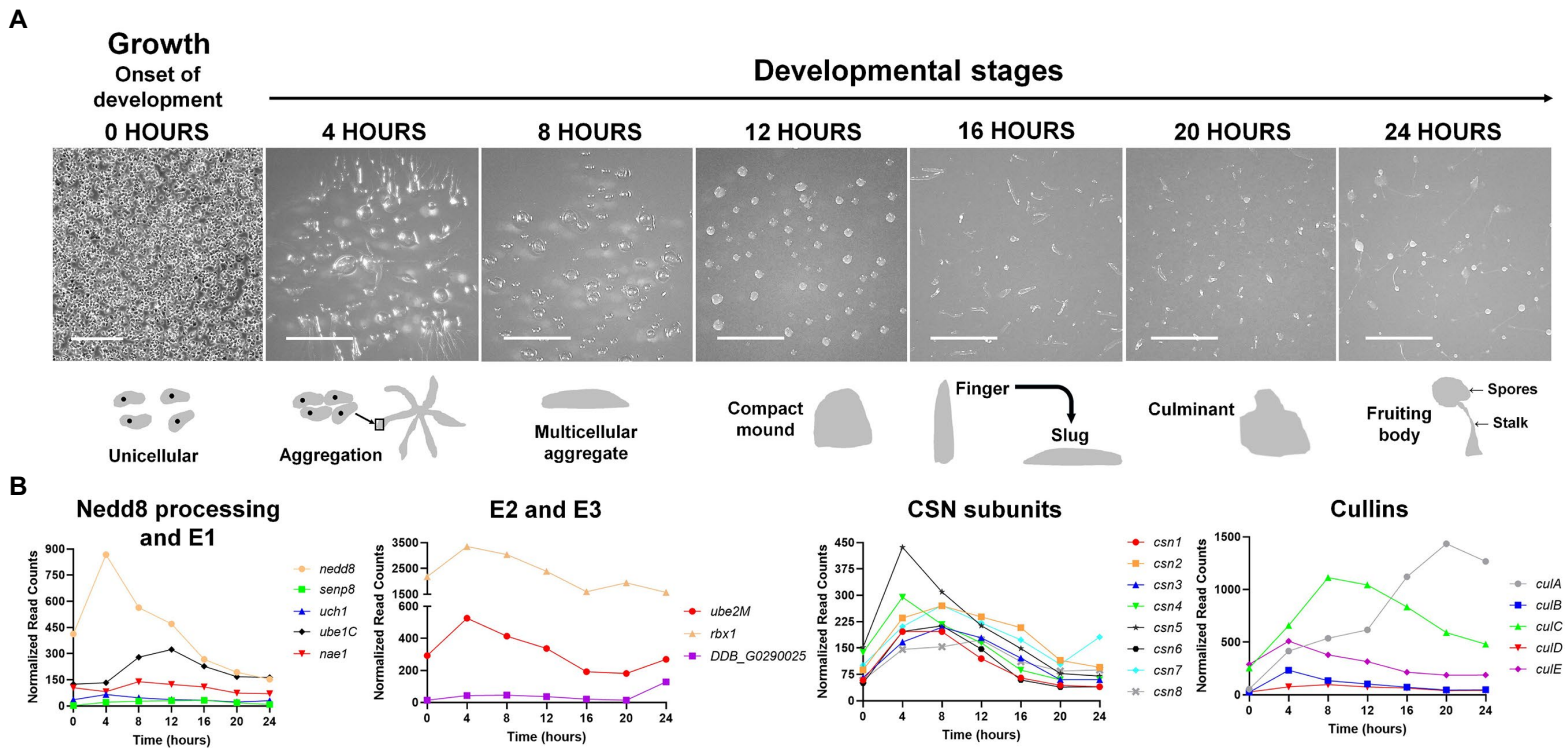
**(A)** The *Dictyostelium discoideum* life cycle. The asexual life cycle of *D. discoideum* occurs within 24 h. *D. discoideum* cells are unicellular during growth and divide by mitosis. Starvation triggers the onset of development. Starved amoebae undergo cyclic adenosine monophosphate (cAMP)-mediated chemotactic aggregation to form a multicellular aggregate, followed by compact mound, which then undergoes a series of morphological changes to form a finger that falls on the surface to generate a motile slug. Cells within the slug then terminally differentiate during culmination to form a fruiting body composed of a mass of viable spores that sit atop a slender stalk. In the laboratory, the multicellular developmental program is induced by depositing cells on non-nutrient agar plates. Scale bar (0 h) = 200 μm. Scale bar (4–24 h) = 2 mm. Images are not drawn to scale. **(B)** Expression profiles of cullin genes, genes encoding CSN subunits, and genes involved in Nedd8 processing, activation, and conjugation during the *D. discoideum* life cycle. Transcript data were derived from RNA-Seq data obtained from dictyExpress (http://www.dictyexpress.biolab.si) and replotted using GraphPad Prism 8. Expression profiles were examined for genes that encode proteins involved in Nedd8 processing (*nedd8*, *senp8*, and *uch1*), E1 (*nae1* and *ube1C*), E2 (*ube2M*), E3 (*rbx1*, *DDB_G0290025*), and the COP9 signalosome (*csn1-8*). Expression profiles for genes that encode cullins (*culA*-*culE*) are also shown. csn, COP9 signalosome; cul, cullin; nae1, Nedd8-activating enzyme E1 subunit 1; nedd8, neural precursor cell expressed developmentally downregulated protein 8; rbx1, ring box 1/regulator of cullins 1; senp8, sentrin-specific protease 8; ube, ubiquitin-activating enzyme; and uch, ubiquitin C-terminal hydrolase.

*D. discoideum* belongs to a clade within the Amoebozoan known as the social amoebae, a term coined by Bonner (1949) after observing that unicellular *D. discoideum* amoebae could develop into multicellular fruiting bodies when prompted by starvation. In the 24-h asexual life cycle of *D. discoideum*, a starved population of amoebae aggregate to form complex multicellular structures in a time-dependent manner (Fey et al., 2007; Gaudet et al., 2008; Mathavarajah et al., 2017; Figure 3A). After aggregating to form a mound, cells rise above the surface to form a finger, which then falls on the surface to form a motile pseudoplasmodium, or slug (Raper, 1940; Brenner, 1977). As multicellular development continues, the slug forms a culminant where cells undergo terminal differentiation to form a fruiting body, the final stage of development (Marée and Hogeweg, 2001). The fruiting body is composed of a mass of differentiated spores that sit atop a slender stalk of differentiated stalk cells. During differentiation, ~80% of the cells within the slug become pre-spore cells, which eventually differentiate into spores (Forman and Garrod, 1977). The other ~20% differentiate into pre-stalk cells and become non-reproductive cells that comprise the stalk and two other segments of the fruiting body. These two other segments are composed of differentiated cells derived from the same cell type as the stalk cells and are referred to as the cup and basal disk cells (they constitute both the cup and basal structures of the fruiting body, respectively; Chen and Schaap, 2016). The *D. discoideum* life cycle highlights the evolution of many processes required for multicellular development, including but not limited to, cell–cell communication, cell–cell adhesion, and differentiation.

## Neddylation, Cullins, and the CSN in *Dictyostelium discoideum*

### Neddylation and Cullins in *Dictyostelium discoideum*

In *D. discoideum*, *nedd8* is expressed throughout development but peaks in expression after 4 h of development (Figure 3B). This expression profile overlaps with the expression profiles of most cullin genes in *D. discoideum*, which peak in expression during aggregation (Figure 3B). Unlike later diverging amorphea, the C-terminal extension is only one amino acid long for *D. discoideum* Nedd8 (Figure 1; Huber et al., 2021). The *D. discoideum* genome also encodes a protein homologous to UCHL1 and UCHL3 in humans, Uch1 (encoded by *uch1*), and an ortholog of human SENP8, Senp8 (encoded by *senp8*; Figure 2; Table 1; Huber et al., 2021).

The *D. discoideum* E1 complex is a heterodimer comprised of orthologs of mammalian NAE1 and UBA3 (Nae1 and Ube1C, respectively; Figure 2; Table 1). There is only one potential E2 encoded by *ube2M* (protein: Ube2M), which is similar in sequence to both UBE2M and UBE2F. The *D. discoideum* genome also encodes an ortholog of RBX1 and RBX2, Rbx1, that is proposed to function as an E3 in the organism, as well as an ortholog of DCUN1D1 (uncharacterized protein DDB0305617; Forman and Garrod, 1977). Finally, *D. discoideum* contains an ortholog of human CAND1 (Cand1). Together, the predicted neddylation pathway in *D. discoideum* shares similarities to the well-established pathway in metazoans.

**Figure 4:**
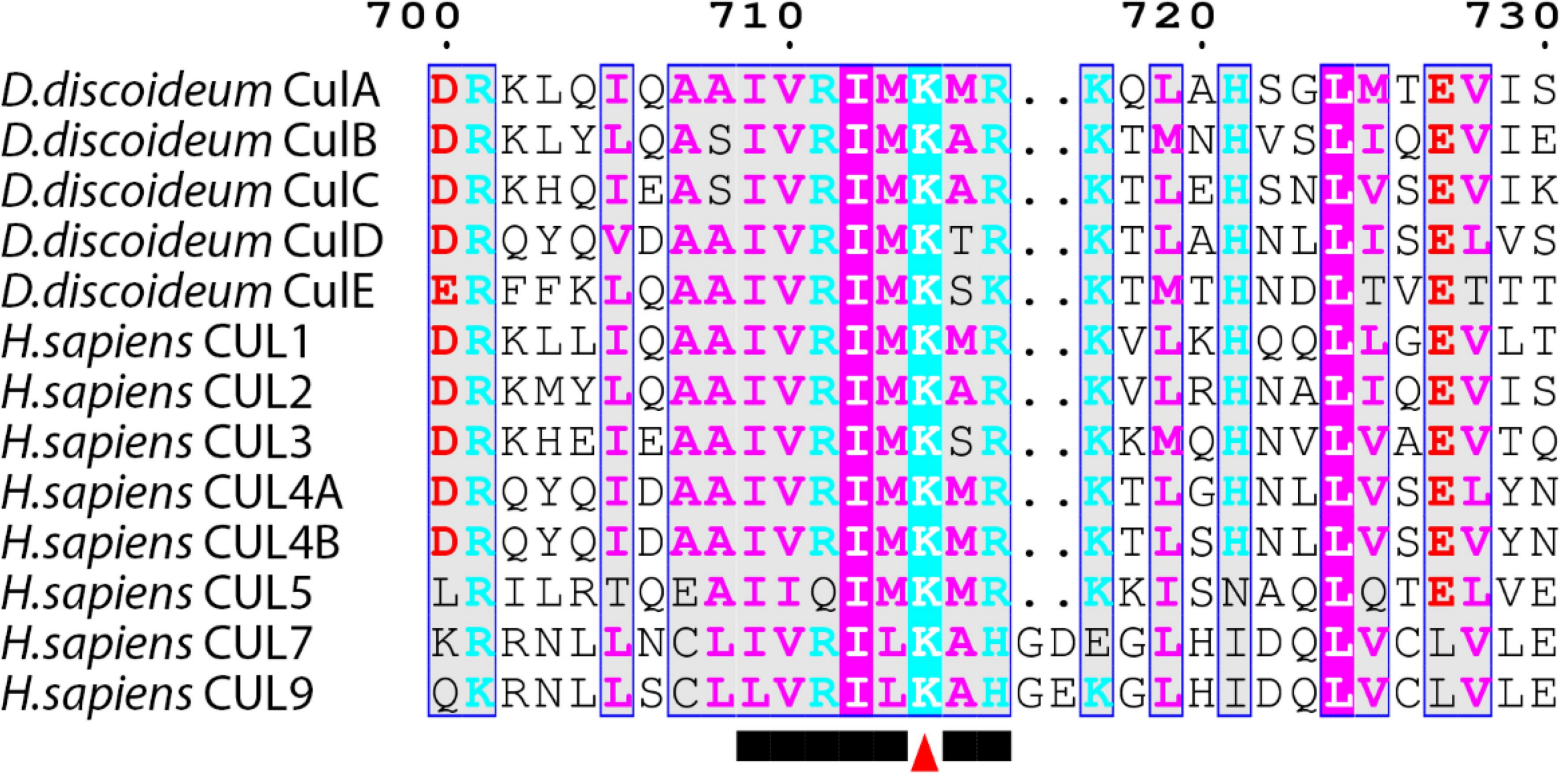
Alignment of cullins from *Dictyostelium discoideum* and human. Sequences were aligned on MEGA7 using the MUSCLE alignment and displayed using ESPript 3.0. Colored residues indicate physiochemical properties and conservation at a position. Arrowhead marks the lysine motif where the NEDD8 protein is conjugated within the conserved motif.

In mammals, there are eight members of the cullin family (CUL1, CUL2, CUL3, CUL4A, CUL4B, CUL5, CUL7, and CUL9; Table 2; Sarikas et al., 2011). The *D. discoideum* genome encodes five proteins (CulA, CulB, CulC, CulD, and CulE encoded by *culA*, *culB*, *culC*, *culD*, and *culE*, respectively) that all share sequence similarity with human cullins (Table 2). Moreover, Sheikh et al. (2015) compared characteristic sequence motifs in cullins to show that CUL1 is most similar to CulA, CulE, and CulB, CUL3 is most similar to CulC, and CUL4B is most similar to CulD. BLASTp searches also show that the *D. discoideum* ortholog of anaphase promoting complex subunit 2 (Anapc2) shares limited sequence similarity with human CUL1, CUL2, CUL3, CUL4A, and CUL4B (Table 2). In mammals, anaphase promoting complex functions as an E3 ubiquitin ligase that regulates cell cycle progression by mediating ubiquitination and subsequent degradation of target proteins (Tang et al., 2001; Jin et al., 2008). Since only CulE has previously been validated as a neddylated protein, we examined the sequences of other *D. discoideum* cullins to determine whether the neddylation motif is conserved (Robert and Gouet, 2014; Kumar et al., 2016). The alignment revealed that the cullin neddylation motif is highly conserved between cullins from *D. discoideum* and humans (Figure 4). While the residues upstream of the lysine are identical and conserved in *D. discoideum* cullins, there are differences in the two downstream residues. Adjacent to the lysine (+1 position), CulD has a threonine rather than adhering to the (MAS) amino acid sequence (methionine, alanine, or serine at the +1 position), indicating functional flexibility between the threonine and serine groups in *D. discoideum*. Similarly, CulE differs at the +2 position with a lysine residue that normally contains either an arginine, histidine, or glutamic acid residue [i.e., (RHE)]. Since there is a highly conserved neddylation motif present in each of CulA, CulB, CulC, CulD, and CulE, there is potential for Nedd8 to be conjugated to all *D. discoideum* cullins. Finally, the *D. discoideum* genome encodes orthologs of eight human CSN subunits (Table 1).

**Table 2:** Sequence similarity between cullins in humans and *Dictyostelium discoideum*.

Human protein (Uniprot ID)	Size (aa)	*Dictyostelium discoideum* protein (dictyBase gene ID)	Size (aa)	Region of similarity (aa)	Identities (%)^*^	Positives (%)^**^	E-value
CUL1 (Q13616)	776	CulA (DDB_G0291972)	770	772	51	68	0
		CulB (DDB_G0267384)	771	726	38	61	1E-136
		CulE (DDB_G0278991)	750	783	31	54	1E-104
		CulC (DDB_G0284903)	769	801	30	50	6E-99
		CulD (DDB_G0292794)	802	801	28	47	4E-69
		Anapc2 (DDB_G0276377)	907	226	22	41	6E-05
CUL2 (Q13617)	745	CulA (DDB_G0291972)	770	774	37	57	1E-135
		CulB (DDB_G0267384)	771	777	35	57	1E-131
		CulC (DDB_G0284903)	769	780	29	51	1E-87
		CulE (DDB_G0278991)	750	760	30	51	1E-83
		CulD (DDB_G0292794)	802	780	25	47	3E-63
		Anapc2 (DDB_G0276377)	907	157	24	40	0.013
CUL3 (Q13618)	768	CulC (DDB_G0284903)	769	777	48	66	0
		CulD (DDB_G0292794)	802	790	34	54	1E-113
		CulA (DDB_G0291972)	770	725	32	52	2E-97
		CulB (DDB_G0267384)	771	795	29	51	2E-86
		CulE (DDB_G0278991)	750	704	28	47	2E-62
		Anapc2 (DDB_G0276377)	907	224	22	38	0.25
CUL4A (Q13619)	759	CulD (DDB_G0292794)	802	763	46	64	0
		CulC (DDB_G0284903)	769	776	38	59	1E-142
		CulA (DDB_G0291972)	770	700	33	53	2E-88
		CulB (DDB_G0267384)	771	741	27	49	2E-71
		CulE (DDB_G0278991)	750	611	30	50	2E-61
		Anapc2 (DDB_G0276377)	907	177	25	40	2E-04
CUL4B (Q13620)	913	CulD (DDB_G0292794)	802	761	48	65	0
		CulC (DDB_G0284903)	769	766	38	60	1E-139
		CulA (DDB_G0291972)	770	701	32	54	8E-88
		CulB (DDB_G0267384)	771	741	26	48	7E-67
		CulE (DDB_G0278991)	750	610	29	50	5E-59
		Anapc2 (DDB_G0276377)	907	180	27	40	9E-05
CUL5 (Q93034)	780	CulA (DDB_G0291972)	770	797	28	52	3E-85
		CulB (DDB_G0267384)	771	764	29	51	6E-84
		CulE (DDB_G0278991)	750	661	28	52	3E-65
		CulC (DDB_G0284903)	769	689	26	48	8E-55
		CulD (DDB_G0292794)	802	674	26	47	1E-39
CUL7 (Q14999)	1,698	CulA (DDB_G0291972)	770	358	21	37	0.54
CUL9 (Q8IWT3)	2,517	CulA (DDB_G0291972)	770	391	23	39	4E-05

BLASTp searches were performed using dictyBase. Human proteins were used as query sequences (E-value, 1; Matrix, BLOSUM62; Filter, no).

Sheikh et al. (2015) performed a similar analysis to show that CUL1 is most similar to CulA, CulE, and CulB, CUL3 is most similar to CulC, and CUL4B is most similar to CulD.

* Exact amino acid match.

** Similar amino acid match (e.g., both polar).

### Cullin Gene Expression During the *Dictyostelium discoideum* Life Cycle

The *D. discoideum* cullin genes are differentially expressed during development and peak in expression at different times during the developmental program (Figure 3B). The expression of *culB*, *culC*, *culD*, and *culE* increase and reach peak levels during the first 8 h of development, followed by a decline throughout the remaining stages of development. Conversely, *culA* rises in expression throughout development and reaches its peak level after 20 h. Consistent with this, Mohanty et al. (2001) used an anti-CUL1 antibody to show that a cullin (presumed to be CulA) reaches peak levels after 16–20 h of development. Combined, these findings suggest that cullins may have specific roles during *D. discoideum* development.

### The CulE Interactome in *Dictyostelium discoideum*

**Figure 5:**
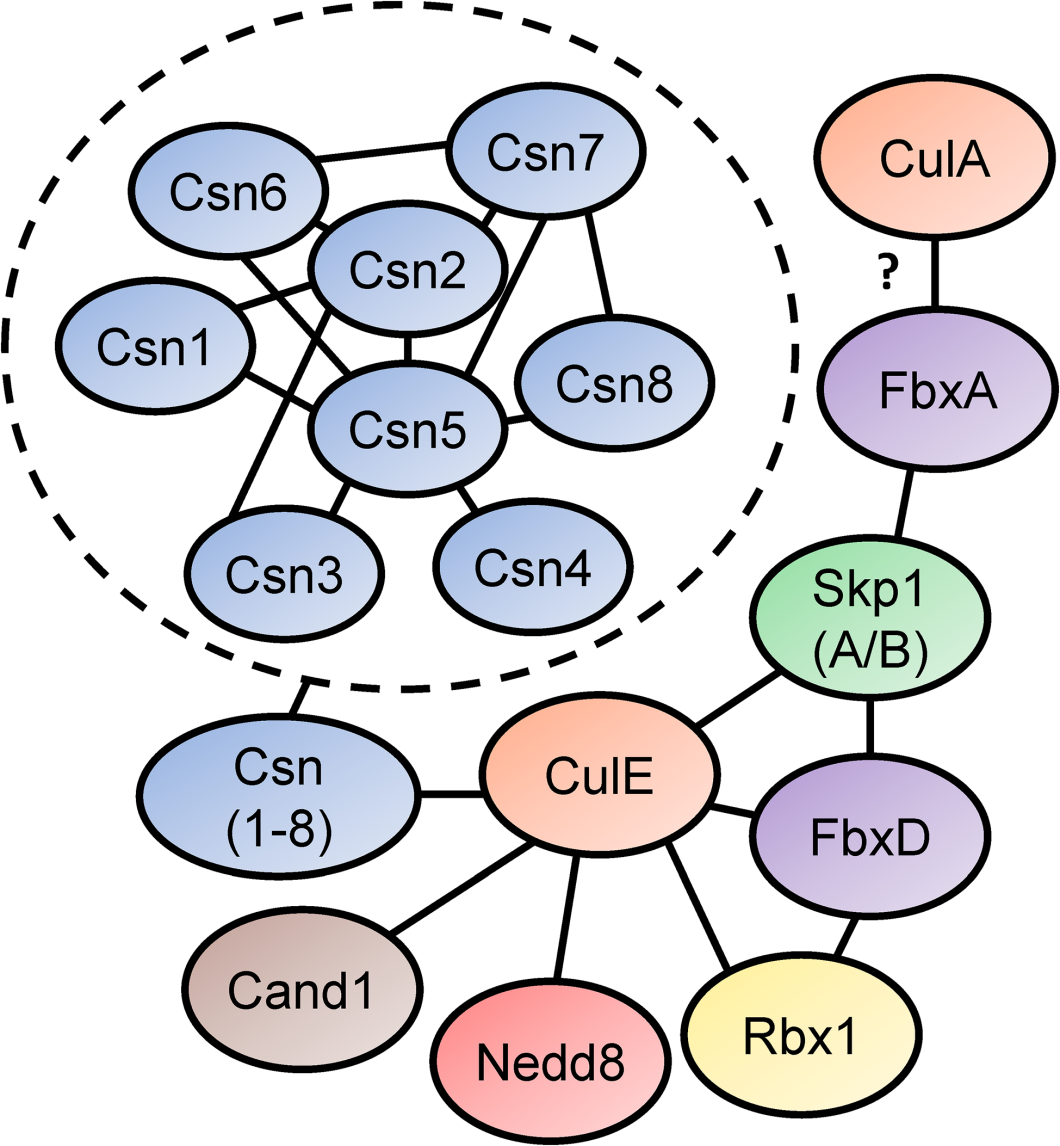
Skp1-Cullin-F-box (SCF) complex interactome reveals components of the neddylation pathway and subunits of the CSN. Sheikh et al. (2015) identified proteins that interact with *D. discoideum* Skp1, FbxD, and CulE after performing co-immunoprecipitations for each protein. Interactors were identified using LC–MS/MS. Connecting lines indicate the interactions between the proteins. Mohanty et al. (2001) reported an interaction between a *Dictyostelium discoideum* cullin (presumably CulA) and FbxA. In the dashed circle, CSN subunit interactions with one another are shown. This was determined in a yeast two-hybrid screen by Rosel and Kimmel (2006). Cand1, cullin-associated Nedd8-dissociated protein 1; Csn, COP9 signalosome; Cul, cullin; Fbx, F-box protein; Nedd8, neural precursor cell expressed developmentally downregulated protein 8; Rbx1, ring box 1/regulator of cullins 1; and Skp1, S phase kinase-associated protein 1.

In Sheikh et al. (2015) identified the SCF interactome in *D. discoideum* by expressing FLAG-tagged CulE in amoebae (Figure 5). In the interactome, proteins orthologous to components of the mammalian SCF complex were identified, such as homologs of SKP1 and three F-box proteins (FbxD, uncharacterized protein DDB0306343, and uncharacterized protein DDB0237864). Using an antibody directed against *D. discoideum* Skp1, another co-immunoprecipitation was performed that identified CulE as a Skp1-interactor via Western blotting (Sheikh et al., 2015). F-box proteins have been shown to interact with cullins in *D. discoideum* (Mohanty et al., 2001; Sheikh et al., 2015). For example, FbxD has been identified as a CulE interactor (discussed above; Sheikh et al., 2015; Figure 5). In addition, Mohanty et al. (2001) reported an *in vivo* interaction between CulA and FbxA using an antibody against human CUL1 that failed to detect a protein in *culA^−^* cells via Western blotting. However, since the antibody was not specific for CulA, and there is strong sequence similarity between cullins in *D. discoideum* (and humans; Table 2; Sheikh et al., 2015), it is possible that another cullin was detected in the FbxA pull-down. Nonetheless, these observations suggest that the SCF complex associates with distinct cullins at different points in the life cycle to regulate specific processes during multicellular development. In addition, Skp1 modification affects the representation of F-box proteins in the Skp1 interactome suggesting that it influences the recruitment of F box proteins to the SCF complex (Sheikh et al., 2015).

The CulE interactome also revealed that known mechanisms of SCF regulation are conserved in *D. discoideum* (Enchev et al., 2015). For example, an ortholog of mammalian CAND1 (Cand1) was identified as an interactor suggesting that the mechanisms regulating CRL assembly and disassembly are similar in *D. discoideum* to what is observed in other eukaryotes (Figure 5). Furthermore, CulE was shown to interact with several proteins involved in neddylation and deneddylation such as Nedd8, Rbx1, and all the Csn subunits (Sheikh et al., 2015). As additional support for the neddylation of CulE, Western blotting for the CulE protein shows two distinct protein bands (Sheikh et al., 2015). In total, accumulated evidence strongly supports that not only is the neddylation machinery conserved in *D. discoideum*, but it also dynamically regulates the assembly of SCF complexes in *D. discoideum* by acting on cullins.

## The Roles of Cullins and Their Regulation During *Dictyostelium discoideum* Growth

### Roles of Neddylation and the CSN in Cell Proliferation

**Figure 6:**
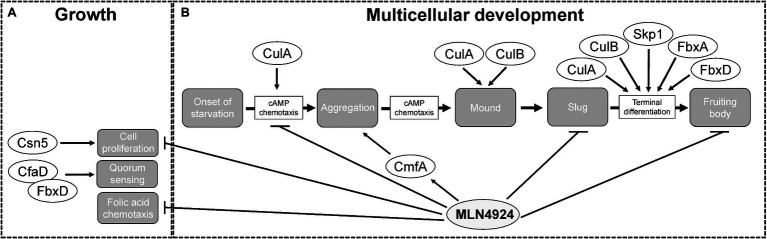
The roles of cullins, neddylation, and the CSN during the *Dictyostelium discoideum* life cycle. **(A)** During growth, MLN4924 inhibits cell proliferation and folic acid-mediated chemotaxis. Loss of *csn5* also inhibits cell proliferation. FbxD binds CfaD, which plays a role in quorum sensing. **(B)** During multicellular development, MLN4924 and loss of *culA* inhibit cAMP-mediated chemotaxis, which delays aggregation. MLN4924 also affects the intracellular and extracellular levels of CmfA, which plays a role in quorum sensing during aggregation. *culA^−^* and *culB^−^* cells form multi-tipped mounds during development, which is characteristic of *D. discoideum* mutants with defects in the autophagy pathway. MLN4924 inhibits slug and fruiting body formation. CulA, CulB, Skp1, FbxA, and FbxD collectively regulate fruiting body formation. CfaD, counting factor-associated protein A; CmfA, conditioned media factor A; Csn5 and COP9 signalosome complex subunit 5; Cul, cullin; Fbx, F-box protein; and Skp1, S phase kinase-associated protein 1.

While the role of neddylation is well established for Opisthokonta (major clade containing both fungi and animals), until recently, it was unknown whether neddylation regulates the life cycle of organisms belonging to Amoebozoa (sister group to Opisthokonta; Lammer et al., 1998; Liakopoulos et al., 1998; Gan-Erdene et al., 2003; Mendoza et al., 2003; Wu et al., 2003; Shen et al., 2005; Chan et al., 2008; Shin et al., 2011). Recent work used the well-established NAE1 inhibitor, MLN4924 (Pevonedistat), to reveal the roles of neddylation during *D. discoideum* growth and multicellular development (Huber et al., 2021). In Opisthokonta, neddylation regulates cell cycle progression and consequently, cell proliferation (Lammer et al., 1998; Liakopoulos et al., 1998; Gan-Erdene et al., 2003; Mendoza et al., 2003; Wu et al., 2003; Shen et al., 2005; Chan et al., 2008; Shin et al., 2011). In *D. discoideum*, MLN4924 significantly reduces cell proliferation in a dose-dependent manner during the growth phase of the life cycle (Figure 6; Huber et al., 2021). In addition, counting factor-associated protein D (CfaD), which is a secreted quorum sensing protein that modulates cell proliferation, was detected in the FbxD interactome (Figure 6; Bakthavatsalam et al., 2008; Sheikh et al., 2015). These findings indicate a conserved role for neddylation in regulating cell proliferation and support recent work linking neddylation to the proliferation of cancer cells (Du et al., 2021; Zhang et al., 2021). However, whether MLN4924 specifically affects CRLs in *D. discoideum* remains to be determined since non-cullin targets of neddylation have been reported in several organisms including *S. pombe*, *A. thaliana*, and *Trypanosoma brucei* (Girdwood et al., 2012; Enchev et al., 2015; Mergner et al., 2015; Liao et al., 2017).

Deneddylation of cullins occurs through the actions of SENP8 and the CSN. Deneddylation by the CSN occurs via the CSN5 subunit, which is a metalloprotease (Cope et al., 2002; Echalier et al., 2013). For that reason, CSN5 has the highest selection pressure and is the most conserved of all the CSN subunits. Thus, in *D. discoideum*, Csn5 would be considered the essential subunit of the CSN required for the deneddylation of cullins. Consistent with this, loss of *csn5* impairs cell proliferation in *D. discoideum* (Figure 6; Rosel and Kimmel, 2006). Since reduced neddylation and impaired deneddylation both impact cell proliferation *D. discoideum*, these findings indicate that cell proliferation in *D. discoideum* requires efficient cycling of neddylation and deneddylation.

### Role of Neddylation in Folic Acid-Mediated Chemotaxis

*D. discoideum* cells use chemoattractants to sense nutrient levels within the environment. During the growth or feeding stage of the life cycle, *D. discoideum* cells sense and undergo chemotaxis towards folic acid, which is a biomolecule secreted by bacteria (Gerisch, 1982). Our group observed reduced chemotaxis toward folic acid when cells were treated with MLN4924 (Figure 6; Huber et al., 2021). Intriguingly, recent work supports a role for neddylation in regulating macrophage chemotaxis in chronic pancreatitis (Lin et al., 2021). Therefore, work in *D. discoideum* and humans indicates that neddylation plays an important regulatory role in chemotaxis. As a result, further probing of the chemotactic pathway in *D. discoideum* may improve our understanding of the mechanisms that CRLs use to regulate chemotaxis in humans.

## The Role of Cullins and Their Regulation During *Dictyostelium discoideum* Early Development

### CulA Is Required for cAMP-Mediated Chemotaxis

During the early stages of multicellular development (0–10 h), *D. discoideum* amoebae aggregate by chemotaxis toward cyclic adenosine monophosphate (cAMP), which is produced and secreted by starving cells (Figure 3A; Almeida and Dilao, 2016; Nakajima and Sawai, 2016). Intriguingly, several cullin genes increase their expression during this stage of the life cycle (Figure 3B). Consistent with this, loss of *culA* delays aggregation (Figure 6; Mohanty et al., 2001). Using an assay that examines chemotaxis competence, Mohanty et al. (2001) showed that the response of *culA*^−^ cells to cAMP was suboptimal suggesting that CulA has an early role in development by influencing cAMP-mediated chemotaxis during aggregation (Noegel et al., 1986). As discussed above, the role of neddylation in regulating chemotaxis in *D. discoideum* is consistent with its role in regulating macrophage chemotaxis in chronic pancreatitis (Lin et al., 2021).

In *D. discoideum*, cAMP controls the actions of cAMP-dependent protein kinase A (PKA), which is a signaling kinase that regulates the expression of genes required for inducing aggregation, cell-type differentiation, and culmination (Loomis, 1998). Upstream of PKA activation, the cAMP phosphodiesterase, RegA, catalyzes the conversion of cAMP to 5′-adenosine monophosphate to prevent PKA activation (Shaulsky et al., 1996, 1998; Thomason et al., 1998). As a result of its regulatory role during aggregation, the level of RegA protein peaks at this stage of the developmental program (Mohanty et al., 2001). However, loss of *culA* or *fbxA* causes the level of RegA protein to remain high even after aggregation (Mohanty et al., 2001; Tekinay et al., 2003). When *culA^−^* cells express a constitutively active PKA protein, the observed defects in aggregation and chemotaxis are absent (Mohanty et al., 2001). Since constitutively active PKA functions irrespective of cAMP levels, this supports the notion that abnormally high amounts of RegA interfere with cAMP levels to dysregulate PKA activity (Zhang et al., 2003). In conclusion, there is a significant role for CulA, and the SCF complex it functions with, in early development where it regulates the ubiquitination and degradation of RegA to maintain intracellular cAMP levels for PKA activation.

The SCF-dependent ubiquitination of RegA also appears to be dependent on extracellular signal-regulated kinase (ErkA), an ortholog of mammalian mitogen-activated protein kinase (MAPK). In *D. discoideum*, ErkA phosphorylates RegA to inhibit its activity and this phosphorylation could then prime RegA for SCF-mediated ubiquitination of RegA (Maeda et al., 2004; Kuburich et al., 2019). Additional work is required to determine if ErkA also directly regulates components of the SCF complex in *D. discoideum*. Intriguingly, ectopic expression of mouse *Cul1* restored RegA degradation in *culA^−^* cells suggesting that the functions of mouse CUL1 and *D. discoideum* CulA are conserved (Mohanty et al., 2001).

### Role of Neddylation During Aggregation

During *D. discoideum* development, cullins are differentially regulated and this is possibly linked to their roles in developmental processes at specific time points. Most cullin genes (except *culA*) peak in expression during the first 12 h of development when cells aggregate to form multicellular mounds (Figure 3B). In addition, genes involved in the conjugation of Nedd8 to cullins such as *nedd8*, *uch1*, *nae1*, *ube1C*, *ube2M*, and *rbx1* all follow a similar pattern of expression to the cullin genes, where they peak in expression during the first 12 h of development and then decline in expression throughout the remaining stages (Figure 3B). These data suggest that transcriptional changes in genes associated with neddylation occur in tandem with changes in the expression of cullin genes. Since neddylation promotes CRL assembly, this would allow for timely assembly of CRLs early in development to mediate the initiation of development and aggregation. In addition, CRL assembly and disassembly are thought to occur at least as frequently as new substrate selection (Kleiger et al., 2009; Deol et al., 2019). Consistent with this, the CSN subunits in *D. discoideum* also peak in expression during the first 12 h of development (Figure 3B). Combined, these results indicate that CRL assembly and disassembly are regulated by cycles of neddylation and deneddylation and that this cycling plays an important role in regulating the early stages of *D. discoideum* development.

Treatment of *D. discoideum* cells with MLN4924 delayed aggregation in a dose-dependent manner (Figure 6; Huber et al., 2021). Moreover, this delay persisted even after 24 h, where cells were still unable to form compact mounds (Huber et al., 2021). Similarly, loss of *culA* was shown to impact cAMP-mediated chemotaxis and delay aggregation (Figure 6; Mohanty et al., 2001). Since neddylation inhibition phenocopies this result, it suggests that SCF complex assembly is inhibited when neddylation does not occur, which is consistent with observations in human cells (Zheng et al., 2002; Dubiel et al., 2013). These results also reveal that by upregulating the expression of *nedd8* and cullins during cell aggregation, *D. discoideum* utilizes neddylation as a mechanism to facilitate the timely assembly of the SCF complex. Therefore, the mechanism underlying how neddylation influences cell aggregation is likely tied to its regulation of the SCF complex. Finally, MLN4924 also affects the secretion of the quorum sensing protein conditioned medium factor A (CmfA) during aggregation (Figure 6; Huber et al., 2021). Upon starvation, amoebae begin secreting CmfA, which acts as a trigger for gene expression, as a high density of starved cells correlates to a high concentration of CmfA (Loomis, 2014). Once a CmfA threshold is reached, cells upregulate the expression of developmental genes, including spore coat protein (*cotB*) and cysteine protease D (*cprD*), and aggregate through cAMP-mediated chemotaxis (Loomis, 2014). Inhibition of neddylation by MLN4924 increases the intracellular and extracellular amounts of CmfA (Huber et al., 2021). While CmfA was not identified as an interactor of the *D. discoideum* SCF complex (Sheikh et al., 2015), the effect of MLN4924 on its intracellular and extracellular levels suggests it is regulated by neddylation.

## The Roles of Cullins and Their Regulation During the Mid-to-Late Stages of *Dictyostelium discoideum* Development

### Roles of Cullins and Their Regulation During Slug Formation and Migration

A potential explanation for how cullins influence cell-type differentiation involves the process of autophagy. Autophagy is a metabolic pathway that degrades intracellular material through lysosomal digestion (Mizushima et al., 2008). Autophagy is a required pathway for *D. discoideum* development and influences cell differentiation (Otto et al., 2003). In mammalian cells, the roles of cullins in regulating autophagy are well-established and occur at different steps in the autophagy pathway (McEwan and Dikic, 2014; Mesquita et al., 2017). In general, cullins regulate the ubiquitination of proteins belonging to the autophagy machinery, mTOR activation, and the activation transcription factors.

There also appears to be a link between cullins and autophagy in *D. discoideum*. During the transition from mounds to slugs, mounds form a single tip (Figure 3A). However, *culA^−^* and *culB^−^* cells form multi-tipped mounds during development, which is characteristic of *D. discoideum* mutants with defects in the autophagy pathway (Figure 6; Mohanty et al., 2001; Wang and Kuspa, 2002; Otto et al., 2003; Mesquita et al., 2017). These results indicate that cullins may influence autophagy in *D. discoideum* thereby impacting cell differentiation. However, at present, this link remains to be experimentally determined for the *D. discoideum* cullins.

### Role of the SCF Complex in Oxygen Sensing

Chemical and physical cues from the environment (e.g., light, temperature, and ammonia) are critical for regulating *D. discoideum* development, an organism normally found in soils worldwide (Bonner and Lamont, 2005; Singleton et al., 2006; West et al., 2007; Mathavarajah et al., 2017). Accessible oxygen influences slug polarity and migration, cell differentiation, and other aspects of multicellular development (Sternfeld and Bonner, 1977; Sternfeld and David, 1981; Sternfeld, 1988; Sawada et al., 1998; Bonner, 2003; Biondo et al., 2021; Cochet-Escartin et al., 2021). However, the way this oxygen is sensed by *D. discoideum* differs from what occurs in metazoans. Before the evolution of metazoans, single-celled eukaryotes like *D. discoideum* and *Toxoplasma gondii* lacked the hypoxia inducible factor system for oxygen sensing (Liu, 2017). Instead, these protozoans relied on modifying the SCF complex to mediate oxygen sensing and the mechanism behind this has been well studied in *D. discoideum* (reviewed in West et al., 2010). In *D. discoideum*, regulation of the SCF complex allows for oxygen sensing and this occurs via post-translational modifications. Skp1 of the SCF complex is post-translationally modified at Pro143 through prolyl hydroxylation (the addition of a hydroxyl group) via the prolyl hydroxylase gene (*phyA*; West et al., 2010). Since *phyA* is a direct oxygen sensor, the presence of oxygen functions as the initial stimuli for Skp1 hydroxylation. Following hydroxylation, Skp1 is O-glycosylated through the actions of several glycosyltransferases including GlcNAc transferase (GntA), poly-glycosyltransferase (PgtA), and alpha-gal-transferase (AgtA; in that order; Teng-Umnuay et al., 1999; van der Wel et al., 2001; Ketcham et al., 2004). Glycosylation then promotes the association of Skp1 with F-box proteins and allows for the rapid assembly of the SCF complex to regulate culmination and spore formation, and perhaps other oxygen-dependent developmental pathways (Xu et al., 2012; Sheikh et al., 2015). In conclusion, this represents a novel post-translational modification of the SCF complex that is utilized for oxygen-dependent development in *D. discoideum*.

### Cullins and Neddylation Influence Cell-Type Differentiation

After aggregation, *D. discoideum* cells undergo cell-type differentiation to become pre-spore or pre-stalk cells, which each express cell-specific markers; extracellular matrix protein A (EcmA) for pre-stalk; and spore coat protein 60 (SP60/CotC) for pre-spore (Fosnaugh and Loomis, 1989; Morrison et al., 1994; Williams, 2006). When these markers were examined post-aggregation in *culA*^−^ cells, *ecmA* expression was absent and *cotC* expression was decreased (Mohanty et al., 2001). In addition, an altered ratio of pre-stalk to pre-spore cells was reported in *fbxA*^−^ cells and overexpression of FLAG-tagged FbxD has been shown to delay fruiting body formation (Nelson et al., 2000; Sheikh et al., 2015). Finally, inhibiting neddylation with MLN4924 was shown to impair fruiting body formation (Huber et al., 2021). Collectively, these findings indicate that CulA, FbxA, FbxD, and neddylation have roles in cell differentiation.

Cullin genes in *D. discoideum* have selective roles in cell differentiation. Unlike what was seen with loss of *culA*, *culB^−^* cells express *ecmA* precociously and have a propensity to form pre-stalk cells (Wang and Kuspa, 2002). Moreover, *culB*^−^ cells that express constitutively active PKA differentiate into stalk cells prior to even reaching the mound stage of development (Wang and Kuspa, 2002). In addition, PKA activity is antagonistic with the loss of *culB*, worsening the phenotype, and contrasting work with *culA*^−^ cells (Wang and Kuspa, 2002). These results suggest that CulB has non-CRL differentiation functions or is utilized in another unique CRL complex involved in regulating differentiation in *D. discoideum*.

Previous work suggests that cell-type differentiation in *D. discoideum* may also be influenced by *culD* and *culE*. In a recent study examining the expression of genes in different cell-types after differentiation, *culD* and *culE* transcripts were both preferentially upregulated in spores and downregulated in stalk cells (Kin et al., 2018). Altered expression in specific cell types may occur to (1) directly facilitate the terminal differentiation (i.e., pre-stalk to stalk cell) or (2) allow for functions related to the distinct roles of the cell types. Future work examining these two cullin genes will help elucidate how *culD* and *culE* contribute to cell differentiation during multicellular development.

## Conclusion

*D. discoideum* is a well-established model organism that has been studied for close to 100 years (Raper, 1935). Its 24-h life cycle is comprised of unicellular and multicellular phases that allows for a detailed examination of a multitude of fundamental cellular and developmental processes in the context of a whole organism (Mathavarajah et al., 2017). *D. discoideum* can be cultured rapidly and inexpensively at room temperature in liquid medium (8–12 h doubling time) or on non-nutrient agar with bacteria (3–4 h doubling time; Fey et al., 2007). Importantly, *D. discoideum* is genetically tractable and a variety of expression constructs have been generated to facilitate studies on protein localization and function (Levi et al., 2000; Kuspa, 2006; Veltman et al., 2009; Faix et al., 2013; Müller-Taubenberger and Ishikawa-Ankerhold, 2013; Friedrich et al., 2015; Yamashita et al., 2021). For these and other reasons, it has also been used as a high-throughput biomedical model for studying variety of human diseases (Huber, 2021; Kirolos et al., 2021; Mathavarajah et al., 2021; Pain et al., 2021).

Regulated protein degradation is an essential process in all eukaryotes. In *D. discoideum*, CRL-mediated ubiquitination regulates complex processes associated with growth and multicellular development (Figure 6). In addition, inhibiting neddylation with MLN4924 impacts cell proliferation, chemotaxis, aggregation, and multicellular development. As a result, future work in *D. discoideum* has the potential to enhance our understanding of the cellular and developmental roles of cullins, neddylation, and the CSN. For example, cell migration is an important physiological process that occurs during wound healing, embryonic development, and disease (e.g., cancer metastasis). *D. discoideum* is an ideal model system for studying fundamental aspects of cellular migration, particularly the mechanisms underlying chemotaxis (Kamimura and Ueda, 2021; Kuhn et al., 2021; Xu et al., 2021). As discussed in this review, neddylation regulates the migration of a variety of cell types (Park et al., 2018; Kim et al., 2021). Since MLN4924 inhibits cell migration during *D. discoideum* development, and CulA plays an important role in cAMP-mediated chemotaxis, *D. discoideum* can be used to further explore the role of neddylation and cullins in regulating cell migration and chemotaxis in normal and diseased cells. In addition, neddylation has been linked to autophagy regulation in esophageal and liver cancer (Luo et al., 2012; Chen et al., 2015; Liang et al., 2020). Since the mechanisms regulating autophagy in *D. discoideum* are like those that regulate autophagy in mammals (Mesquita et al., 2017), *D. discoideum* can be used to increase our understanding of how neddylation regulates autophagy. Finally, although there are significant differences between metazoan and *D. discoideum* development, studying the roles of neddylation and cullins in regulating multicellular development in *D. discoideum* may uncover conserved developmental roles that can then be validated in mammalian models and humans. This is important since there is a need to better understand the mechanisms regulating timely protein degradation events during metazoan development, as recent studies have reported an essential role for neddylation in cardiac development (Li et al., 2020). Thus, *D. discoideum* can be used as a model system to better understand different aspects of the neddylation pathway and CSN during development, which has implications for several diseases revolving around dysregulated neddylation. Together, this review highlights the use of *D. discoideum* as a model system to better understand the conserved cellular and developmental roles of cullins, neddylation, and the CSN.

## Author Contributions

SM and RJH: conceptualization. WDK and SM: writing—original draft. WDK, SM, and RJH: writing—review and editing. RJH: supervision and funding acquisition. All authors contributed to the article and approved the submitted version.

## Funding

This work was supported by a Discovery Grant from the Natural Sciences and Engineering Research Council of Canada (RGPIN-2018-04855 to RJH). WDK was supported by a Queen Elizabeth II Graduate Scholarship in Science and Technology.

## Conflict of Interest

The authors declare that the research was conducted in the absence of any commercial or financial relationships that could be construed as a potential conflict of interest.

## Publisher’s Note

All claims expressed in this article are solely those of the authors and do not necessarily represent those of their affiliated organizations, or those of the publisher, the editors and the reviewers. Any product that may be evaluated in this article, or claim that may be made by its manufacturer, is not guaranteed or endorsed by the publisher.

## Figures and Tables

**Table:** 

AgtA	Alpha-gal-transferase
Anapc2	Anaphase promoting complex subunit 2
cAMP	Cyclic adenosine monophosphate
CAND1	Cullin-associated NEDD8-dissociated protein 1
cAMP	Cyclic adenosine monophosphate
Cdk	Cyclin-dependent kinase
CfaD	Counting factor-associated protein D
CmfA	Conditioned media factor A
CotC/SP60	Spore coat protein SP60
CRL	Cullin–RING ligase
CSN	COP9 signalosome
CUL	Cullin
DCUN1D1	Defective in cullin neddylation 1
EcmA	Extracellular matrix protein A
ErkA	Extracellular signal-regulated kinase
Fbx	F-box-containing protein
GntA	GlcNAc transferase
MAPK	Mitogen-activated protein kinase
MTOR	Mammalian target of rapamycin
NAE1	NEDD8 activating enzyme E1 regulatory subunit
NEDD8	Neural precursor cell expressed developmentally downregulated protein 8
PARP1	Poly(ADP-ribose) polymerase 1
PgtA	Poly-glycosyltransferase
PhyA	Prolyl hydroxylase
PKA	Protein kinase A
RBX1/ROC1	Ring box 1/regulator of cullins 1
RBX2/ROC2	Ring box 2/regulator of cullins 2
SCF	Skp1-Cullin-F-box
SENP8/DEN1	Sentrin-specific protease 8
SKP1	S phase kinase-associated protein 1
SUMO	Small ubiquitin-like modifier
UBA3	Ubiquitin-activating enzyme 3
UBE2F	Ubiquitin-conjugating enzyme E2F
UBE2M/UBC12	Ubiquitin-conjugating enzyme E2M
UCHL	Ubiquitin C-terminal hydrolase isozyme
UFM1	Ubiquitin fold modifier 1
URM1	Ubiquitin-related modifier-1
